# Long-Term Efficacy and Safety of Direct Oral Anticoagulants at Reduced Doses in the Secondary Prevention of Venous Thromboembolism and Post-Thrombotic Syndrome

**DOI:** 10.3390/jcm13082394

**Published:** 2024-04-19

**Authors:** Luca Costanzo, Federico Di Paola, Anastasia Maria Pedi, Giacomo Failla, Marco Mangiafico

**Affiliations:** 1 Unit of Angiology, Department of Cardio-Thoraco-Vascular, Policlinico ”G. Rodolico-San Marco” University Hospital, University of Catania, 95124 Catania, Italy; fedecut@hotmail.it (F.D.P.); jacomienko@gmail.com (G.F.); 2 Unit of Internal Medicine, Policlinico “G. Rodolico-San Marco”, 95123 Catania, Italy; anastasia46@live.it (A.M.P.); marcomangiafico@hotmail.it (M.M.)

**Keywords:** DOACs, venous thromboembolism, post-thrombotic syndrome, anticoagulant, deep vein thrombosis

## Abstract

**Background**: Anticoagulation for venous thromboembolism (VTE) is required for at least three to six months; however, it is advisable to extend the duration in certain cases, in which case a reduced dose of Direct Oral Anticoagulants (DOACs) may be an option. Our objective was to investigate the efficacy and safety of reduced-dose DOACs in extended anticoagulation treatment compared to full doses. **Methods and Results**: This retrospective single-centre study included 185 patients treated with DOACs for at least 6 months who were divided into two groups: (1) the Full Dose (FD) group (n = 113) and (2) the Reduced Dose (RD) group (n = 72), which included patients treated with Apixaban at 2.5 mg bis in die (BID) and Rivaroxaban at 10 mg once daily (OD). Post-thrombotic syndrome (PTS) and its progression were evaluated. During an overall follow-up of 48.32 ± 29.49 months, no VTE occurred, and no patients experienced major bleeding; clinically relevant non-major bleeding occurred in three patients in each group (2.7% vs. 4.2% in FD vs. RD, respectively, *p* = 0.57). From baseline to follow-up, the prevalence of PTS was not significantly decreased in either group (FD: 54.9% vs. 51.3%, *p* = 0.29; RD 51.4% vs. 44.4%, *p* = 0.12); conversely, the Villalta score values were significantly decreased at the last follow-up (FD: 5.51 ± 4.18 vs. 5.12 ± 4.36, *p* < 0.001; RD 5.49 ± 4.06 vs. 5.11 ± 3.73, *p* = 0.006). **Conclusion:** In this real-world retrospective registry, very long-term extended anticoagulant therapy with DOACs at full or reduced doses showed comparable efficacy, safety, and impact on PTS progression. Larger studies are needed.

## 1. Introduction

Venous thromboembolism (VTE), which clinically includes both deep vein thrombosis (DVT) and pulmonary embolism (PE), is a disease associated with high mortality, morbidity [1,2], and high economic costs for national health systems [3].

European ancestry individuals are estimated to have an annual incidence rate of overall VTE of between 104 and 183 per 100,000 person-years [4].

Increased evidence of VTE was observed among women aged 20–45 and men aged 45–60; therefore, it is primarily a disease of old age [5].

It is estimated that the risk of recurrence of VTE is 10% during the first year, 16% at 2 years, 25% at 5 years, and 36% at 10 years for patients with unprovoked VTE who have completed at least three months of coagulant therapy [6].

VTE events are divided into provoked, where there is a clear trigger for the clot, and unprovoked, where the thrombotic event occurred without an obvious cause [7]; the risk of recurrence is higher in unprovoked events, resulting in different prognostic stratifications and therapeutic approaches [8].

One of the most fearsome sequelae of DVT is the onset of post-thrombotic syndrome (PTS), which is characterized by a broad range of symptoms and signs [9]; it is also burdened by a reduction in patients’ quality of life and increased healthcare costs [10].

The main therapy for VTE is anticoagulant treatment, which consists of heparins, vitamin K antagonists (VKAs), Fondaparinux, and direct oral anticoagulants (DOACs) [11]. According to the latest international guidelines, acute VTE patients should undergo a minimum of 3 months of therapy, and extended therapy is recommended depending on the risk of recurrence. Considering the extension of anticoagulant therapy beyond 6 to 12 months, it is important to balance the haemorrhagic and thrombotic risks, as anticoagulation can cause annual major bleeding (MB) rates of up to 1–2% [12,13,14,15]. In the case of clinical equipoise concerning the continuation or cessation of anticoagulant therapy, a reduced dosage of apixaban (2.5 mg twice daily) and rivaroxaban (10 mg once daily) have shown low rates of VTE recurrence and safety regarding bleeding (Table 1) [16,17].

In the trials mentioned above, Rivaroxaban and Apixaban were administered only for a maximum of 12 months, and there is a small amount of evidence concerning longer treatment in the literature. Therefore, this study aims to evaluate the efficacy and safety of extended treatment with DOACs at reduced doses (r-DOACs) in the secondary prevention of VTE and its impact on PTS clinical evolution.

## 2. Materials and Methods

### 2.1. Study Design, Procedures, and Follow-Up

In our division of Angiology (San Marco Hospital, Catania, Italy), we conducted an observational, non-randomized, retrospective, single-centre study. From May 2017 to February 2024, we retrospectively analyzed all patients with VTE who were treated with DOACs for at least 6 months. We identified two groups: (1) the Full Dose (FD) group and (2) the Reduced Dose (RD) group, in which we included the patients who had been prescribed a reduced dose of Apixaban at 2.5 mg bis in die (BID) and Rivaroxaban at 10 mg once daily (OD) for extended therapy to prevent VTE recurrence, as recommended by current guidelines [12,13]. In our clinic, patients underwent clinical and instrumental follow-ups periodically by one physician. For our analysis, we considered the follow-up as follows: (1) from VTE diagnosis to the last available follow-up for both groups; (2) for the RD group, from VTE diagnosis to the start of the reduced dose, and from the start of the reduced dose until the last available follow-up. PTS was evaluated according to the Villalta score [18]. We reported the presence of PTS and evaluated any changes as follows: in the FD group: (1) six months after the diagnosis of VTE (defined as baseline) and (2) at the last available follow-up; in the RD group: (1) when the reduced dose was started (defined as baseline) and (2) at the last available follow-up. Briefly, PTS was defined as mild if the Villalta score was between 5 and 9, moderate if the Villalta score was between 10 and 14, and severe if the Villalta score was equal to or greater than 15. Additionally, adherence to anticoagulant therapy was periodically evaluated, as in Italy, DOACs are reimbursed and distributed by the National Health System after regular prescription on an online platform of the Italian Agency of Drugs. Therefore, renewing the prescription periodically allowed for regular reassessment of therapeutic indications, which were mainly persistent risk factors, unprovoked DVT, and high risk of recurrence. Good adherence to therapeutic compression stockings was considered as daily wearing for > 5 days and at least 50% during the daytime.

### 2.2. Study Endpoints

The efficacy outcome of the study was the occurrence of death related to VTE and VTE occurrence/recurrence with reduced-dose DOACs compared to full-dose DOACs (RD vs. FD) throughout follow-up. The main safety outcome was the occurrence of MB, defined as a loss in haemoglobin levels of at least 2 g per deciliter, or a loss that led to the transfusion of two or more units of concentrated blood units, or bleeding that occurred in a critical organ and led to the death of the patient. Clinically relevant major bleeding (CRNMB), defined as bleeding that requires medical intervention by a clinician or that leads to hospitalization or otherwise increased levels of patient care, was also evaluated. If the bleeding did not fit the previous definitions, it was defined as “minor”.

PTS was investigated in the FD and RD groups, and the variation of the Villalta score was evaluated at baseline and the last available follow-up.

### 2.3. Statistical Analysis

The Kolmogorov–Smirnov test was used to evaluate the variables for normal distribution. Continuous variables are presented as mean ± standard deviation while dichotomous parameters are presented as frequencies and percentages. Continuous data were compared using the Student’s unpaired *t*-test. Categorical data were compared using the chi-square or Fisher’s test, as appropriate. In case of a non-normal distribution, appropriate non-parametric tests were performed.

All *p*-values were 2-sided, and values of *p* < 0.05 were considered statistically significant. SPSS 20 (IBM SPSS Statistics Base 20, Chicago, IL, USA) was used for statistical analysis.

## 3. Results

The study involved 185 patients—113 in the FD group and 72 in the RD group. Male sex was more prevalent in both groups, with no significant differences (61.9% in FD vs. 55.6% in RD, *p* = 0.44), and patients in the RD group were significantly older (57 ± 15.74 years in FD vs. 64.11 ± 14.47 in RD, *p* < 0.001). The other characteristics of the patients in the two groups were similar (Table 2). In the RD group, Apixaban 2.5 mg BID was prescribed in 24 patients (33.3%) while Rivaroxaban 10 mg OD was administered to 48 patients (66.7%). In the RD group, the average time of the full-dose DOACs treatment before reducing the dose was 28.90 ± 20.29 (range 6 to 96 months, median 24 months).

The overall follow-up was 48.32 ± 29.49 months and a median of 48 months (range 6 to 120 months), being significantly longer in the RD group (59.06 ± 28.74 vs. 41.49 ± 28.00, *p* < 0.001). Notably, in the RD group, the mean follow-up after reducing the DOAC dose was 31.68 ± 17.63 months.

The switch from full dose to r-DOACs occurred for the following reasons: 25 patients (34.7%) due to a high risk of bleeding; 24 patients (33.3%) had clinical equipoise; seven patients (9.7%) had complete recanalization on Doppler ultrasound but also had a history of relapsed VTE, so it was preferred to continue anticoagulant treatment with the reduced dosage; eight patients (11.1%) had a long-standing thrombosis and the reduced dosage was prescribed to lower the bleeding risk; in eight patients (11.1%), the dosage was lowered because bleeding occurred at the DOAC full dose.

Regarding the main efficacy outcome, none of the patients in either group experienced VTE, showing the efficacy of DOACs. No MB occurred. Three patients in each group reported CRNMB (2.7% vs. 4.2% in FD vs. RD, respectively, *p* = 0.57). Minor bleeding occurred in eight (7.1%) of the patients in the FD group and in four (5.6%) of the patients in the RD group (*p* = 0.77).

**Figure 1:**
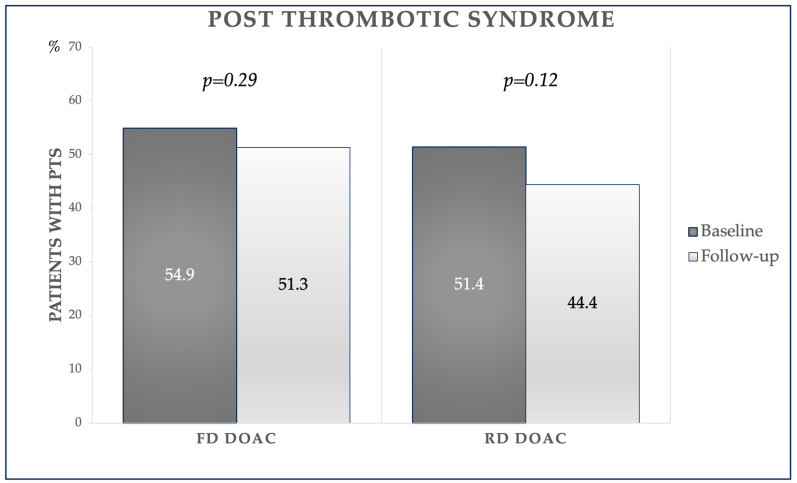
Percentage of patients with post-thrombotic syndrome (PTS) at baseline and follow-up. *DOACs: Direct Oral Anticoagulants*.

**Figure 2:**
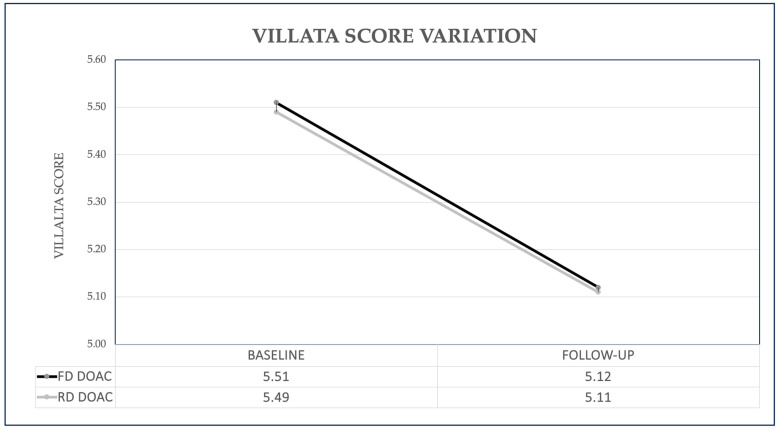
Villalta Score variation in Full Dose (FD) and Reduced Dose (RD) group from baseline to follow-up. *DOACs: Direct Oral Anticoagulants*.

In the FD group, PTS at baseline was found in 62 (54.9%) patients, with a Villalta score of 5.51 ± 4.18; at follow-up, PTS was slightly decreased, being diagnosed in 58 patients (51.3%, *p* = 0.29) of the FD group, with a significant reduction in the Villalta score of 5.12 ± 4.36 (*p* < 0.001). In the RD group, PTS at baseline was diagnosed in 37 (51.4%) patients, with a mean Villalta score of 5.49 ± 4.06. In comparison, at follow-up, the prevalence decreased to 32 patients (44.4%, *p* = 0.12), with a significant reduction in the Villalta score (5.11 ± 3.73 at follow-up, *p* = 0.006). Figure 1 and Figure 2 show the PTS prevalence in both groups and the variation in the Villalta score at baseline and follow-up, respectively.

Notably, all patients had good adherence to the elastic stockings (89.4% in FD and 91.7% RD, *p* = 0.61). Table 3 summarizes the results of the study.

## 4. Discussion

The main findings of our study can be summarized as follows:

(I) Long-term extended therapy for VTE with reduced DOACs was effective and as safe as with a full dose, as none of the patients experienced recurrences, no MB occurred, and CRNMB events were equal. Notably, the average time of treatment with DOACs at full dose before dosage reduction was 24 months, and in about one-third of cases the reason for dosage reduction was due to haemorrhagic phenomena. Additionally, patients in the RD group were significantly older. Therefore, the safety results are reassuring when considering the haemorrhagic risk of the RD population.
(II) The progression of PTS was not negatively impacted by r-DOACs; however, there was even a significant improvement in the Villalta score.

Treatment after an acute VTE event involves at least three months of anticoagulation, whether it is a provoked or unprovoked event. The main determinant of the risk of recurrence after discontinuation of anticoagulation is the aetiology of the first episode of VTE. In patients with VTE caused by a major transient risk factor, treatment may be limited to 3 months, since the risk of recurrence is only 1% per year after discontinuation of anticoagulation [19]. Notably, about half of VTE cases occur without an identifiable risk factor, and in these unprovoked cases, the risk of recurrence after discontinuation of anticoagulation is about 5–10% after one year and 30% after 5 years [19], with a fatality rate of 3.6% [20]. In this category of patients, treatment with VKAs or DOACs beyond three months significantly reduces the risk of recurrence by about 80–90% [21]. Given the high risk of recurrence after discontinuation of anticoagulation, current guidelines advise that all patients with unprovoked VTE or with a persistent major risk factor, without a high risk of bleeding, should continue anticoagulant treatment indefinitely [19]. Conversely, indefinite treatment in all patients with unprovoked VTE exposes a large population to the risk of MB, with a fatality rate of 11.3%, which is two to three-times higher than the recurrent VTE fatality rate [20]. Based on the mortality rates associated with VTE recurrence or MB, the International Society of Thrombosis and Hemostasis (ISTH) suggests that discontinuation of anticoagulation is justified when the annual risk of recurrence is lower than 5% in the first year and 15% in the first 5 years, while it is not justified if the risk of recurrence is 5% in the first year and 30% at 5 years [22].

Therefore, several clinical and laboratory parameters have been evaluated to identify patients who may benefit from indefinite anticoagulation treatment. Among these variables, in a study by Verhovsek et al. [23], the strongest predictors of the risk of recurrence appeared to be sex, the site of VTE, and plasma D-Dimer levels measured 1–2 months after discontinuation of treatment. The analysis of multiple variables led to the creation of prognostic models (such as the Vienna Prediction Model, the DASH score, and the HERDOO2 score). However, these predictive scores have demonstrated substantial methodological limitations and insufficient predictive accuracy [24]. Furthermore, a study conducted by Palareti et al. found that D-dimer measurement was ineffective in identifying patients at low risk of recurrence while evaluating extended treatment [25]. To date, the only validated score able to estimate the absolute risk of recurrence and significant bleeding while on anticoagulation is the VTE-PREDICT score [26], a model derived and validated on a total of 74,398 patients without cancer.

Regarding the use of DOACs in extended treatment, there is a lack of literature data regarding their efficacy and safety in the long term, as well as for the use of r-DOACs.

A large meta-analysis conducted by Khan et al. [27] on the long-term risk of MB during extended treatment with VKA and DOACs showed that the cumulative risk of MB with VKA is considerable after 5 years of therapy (6.3%), but the data are insufficient to estimate the same risk over one year of therapy with DOACs. The same study found a significantly higher incidence of MB in the following populations: age > 65 years, creatinine clearance < 50 mL/min, previous bleeding, haemoglobin levels < 10 g/dL, and concomitant antiplatelet therapy.

MB rates in phase III trials of DOACs for VTE treatment are between 0.1% to 0.9% after 12–15 months of follow-up; however, the rate in the real world may be much higher, as patients at a higher risk of bleeding were excluded from the trials, and the duration of follow-up is limited [28].

In the prospective Xalia and Xalia-LEA studies by Kreutz et al. [29] evaluating the efficacy and safety of rivaroxaban, the rates of MB at one year were 1.2% in XALIA (vs. 3.4% with VKAs) and 1.6% in Xalia LEA (vs. 3.7% in the standard anticoagulation group), with a VTE recurrence of 1.4% (vs. 4% in the standard anticoagulation group). However, no data concerning the use of the reduced 10 mg dose have been reported.

In the Dresden NOAC Registry, the annual rates of CRNMB and MB with rivaroxaban at the standard dose were analyzed in 1776 patients followed from October 2011 to December 2013; MB events were recorded in 4.1% of patients and CRNMB in 17.2% [30].

In a meta-analysis by Bova et al. [31] (six studies—three with VKA and three with DOACs, for a total of 5920 patients), extended anticoagulation treatment was shown to be effective in reducing all causes of mortality (even with Apixaban at a reduced dose) than a shorter course of treatment (0.8% vs. 1.8%, respectively; Relative Risk (RR) 0.47, 95% Confidence Interval (CI) 0.29 to 0.75); the MB rate was not significantly increased in the extended anticoagulation arm (RR 2.29, 95%CI 0.85–6.18), while the rate of non-major bleeding was (RR 2.26, 95%CI 1.52–3.37).

Similar results were provided in the meta-analysis performed by Liu et al. [32], which included 13 randomized controlled trials, and concluded that DOACs were not inferior to standard-intensity warfarin in preventing VTE recurrence with a lower risk of bleeding events. Additionally, the authors identified that apixaban was safer than other DOACs in preventing both MB and CNRMB.

In the meta-analysis by Ebraheem et al. [33], which included three clinical trials with a total of 5021 patients (Amplify-Ext, Einstein-Ext and Re-SONATE), DOACs in extended treatment were proven to reduce the risk of VTE recurrence, but at the same time, there was an increase in CRNMB (RR 2.51, 95%CI 1.37 to 4.59). Additionally, in the meta-analysis by Mai et al. (17 trials, 17,895 patients), DOACs were associated with a reduced risk of MB compared to VKAs, but significance was reached only with Apixaban 5 mg; moreover, in terms of bleeding risk, both apixaban doses were more likely to be the best treatment compared to all other anticoagulants [34].

The first major review and meta-analysis on the efficacy and safety of r-DOACs was published by Vasanthamohan and coworkers [35]. The study included the two largest randomized clinical trials (Amplify Extension and Einstein Choice) of apixaban 2.5 mg BID and rivaroxaban 10 mg OD for a total of 5847 patients. VTE recurrence occurred in 31 of 1967 patients (1.6%) in the r-DOACs group and 27 of 1920 patients (1.4%) in the full-dose DOACs group, with no statistical difference between the two groups, while in the aspirin or placebo group, VTE recurrence was significantly higher (6.3%). Regarding the safety outcomes of MB and CRNMB, events in the r-DOACs group occurred with a similar risk as the aspirin or placebo group (2.3% vs. 2.7%, respectively). In contrast, in the full-dose DOACs group, higher rates of bleeding occurred (3.7%). The authors concluded that r-DOACs were as effective as full-dose DOACs in preventing VTE recurrence at one year, with a similar rate of MB as the aspirin or placebo group. The study emphasized the lack of studies on r-DOACs for extended VTE treatment, with only 12-month follow-up data available; therefore, the safety and efficacy of these drugs after one year are unclear. The current guidelines suggest the use of reduced-dose apixaban or rivaroxaban over full-dose apixaban or rivaroxaban, but this is a weak recommendation with very low certainty evidence [19]. Notably, according to two trials that evaluated a reduced dose of DOACs [16,17], the reduction of DOACs dose after six months was carried out “if there was clinical equipoise about the continuation or cessation of anticoagulant therapy”. Therefore, in case of a clear indication for full-dose anticoagulant (i.e., high risk of recurrence), the dose should not be reduced [15]. Our study, even though retrospective, provides real-world evidence of the efficacy and safety of r-DOACs in the extended treatment of VTE for very long periods.

PTS may develop in 20% to 50% of patients with DVT [36], and our findings confirmed the high prevalence of such a complication. Although PTS may increase in a short time after symptomatic DVT, the cumulative incidence of PTS may continue to increase, even 10 to 20 years after DVT diagnosis [36]. Although our observation time was not so extended, we showed that PTS may improve and that the Villalta score may significantly reduce over time. In our population, the reduction of the DOAC dose did not affect the improvement of PTS over time. Notably, the high adhesion to compression stockings certainly contributed to improving the Villalta score. Therefore, DOACs seem to be effective in preventing the worsening of PTS; however, the mechanism of the DOAC’s positive effect on PTS is still unknown. The hypothesized action could be the prevention of recurrent VTE, the prevention of micro-thrombosis, and positive action on inflammation of the microcirculation [37].

### Study Limitations

The present study has several limitations: First, this was an observational cohort with limitations due to poor control over confounding factors; second, we collected data from a single centre; third, the sample size is small, and this could have affected the outcome rates; fourth, the observation period could be insufficient to adequately assess post-thrombotic syndrome. Fourth, we do not have enough follow-up data on PTS in patients who have discontinued anticoagulant therapy.

## 5. Conclusions

The study demonstrates the safety and effectiveness of treatment with reduced doses of Rivaroxaban (10 mg OD) and Apixaban (2.5 mg BID). The average follow-up was more than 31 months, which showed safety in long-term DOAC therapy in real-world settings. Finally, the data shows that there was a significant reduction in Villalta score PTS values in the RD group.

## Figures and Tables

**Table 1:** Trials testing reduced doses of Apixaban and Rivaroxaban for venous thromboembolism treatment.

	AMPLIFY-EX [16]	EINSTEIN CHOICE [17]
**Number of patients**	2486	3365
**Drug and dose**	Placebo vs. ApiX 2.5 mg BID vs. ApiX 5 mg BID	ASA 100 mg OD vs. RivaX 10 mg OD vs. RivaX 20 mg OD
**Recurrent VTE and VTE-related Death**	Placebo: 8.8%	ASA 100 mg: 4.4%
	ApiX 2.5 mg: 1.7%	RivaX 10 mg: 1.2%
	ApiX 5 mg: 1.7%	RivaX 20 mg: 1.5%
**Major bleeding**	Placebo: 0.5%	ASA 100 mg: 0.3%
	ApiX 2.5 mg: 0.2%	RivaX 10 mg: 0.4%
	ApiX 5 mg: 0.1%	RivaX 20 mg: 0.5%
**Clinically Relevant non-major bleeding**	Placebo: 2.3%	ASA 100 mg: 1.8%
	ApiX 2.5 mg: 3%	RivaX 10 mg: 2.0%
	ApiX 5 mg: 4.2%	RivaX 20 mg: 2.7%
**Recurrent VTE or** **Mortality for all causes**	Placebo: 11.6%	ASA 100 mg: 4.9%
	ApiX 2.5 mg: 3.8%	RivaX 10 mg:1.3%
	ApiX 5 mg: 4.2%	RivaX 20 mg: 2.1%

Legend: ApiX: Apixaban; RivaX: Rivaroxaban; ASA: Aspirin; VTE: Venous Thromboembolism; BID: bis in die; OD: once daily.

**Table 2:** Demographic and Clinical Characteristics of the Study Population.

Demographic and Clinical Characteristics of the Population (n = 185)			
	Therapeutic DOACs (n = 113)	Reduced DOACs (n = 72)	*p*
**Age (years), mean** ± **SD**	57.00 ± 15.74	64.11 ± 14.47	<0.001
**Male sex, n (%)**	70 (61.9)	40 (55.6)	0.44
**BMI, mean** ± **SD**	26.28 ± 4.80	26.86 ± 4.10	0.23
**Recurrent VTE, n (%)**	44 (38.9)	30 (41.7)	0.71
**PE, n. (%)**	28 (24.8)	25 (34.7)	0.18
**Proximal VTE, n (%)**	94 (83.2)	64 (88.9)	0.29
**Provoked thrombosis, n (%)**	23 (20.4)	15 (20.8)	1
**Smokers, n (%)**	37 (32.7)	20 (27.8)	0.52
**Arterial hypertension, n (%)**	53 (46.9)	42 (58.3)	0.13
**eGFR (ml/min), mean** ± **SD**	91.12 ± 21.29	88.99 ± 22.46	0.44
**Active cancer, n (%)**	10 (8.8)	8 (11.1%)	0.61
**Heterozygosity Factor V Leiden, n (%)**	15 (13.3)	9 (12.5)	1
**Heterozygosity Factor II, n (%)**	8 (7.1)	7 (9.7)	0.58
**Protein C deficiency, n (%)**	1 (0.9)	1 (1.4)	1
**Protein S deficiency, n (%)**	24 (21.2)	12 (16.7)	0.46
**Antithrombin III deficiency n (%)**	2 (1.8)	4 (5.6)	0.21

Legend: DOACs: Direct Oral Anticoagulants; n: number; SD: Standard Deviation; BMI: Body mass index; PE: Pulmonary embolism; VTE: Venous thromboembolism; eGFR: Estimated Glomerular Filtration Rate; ApiX: Apixaban; RivaX: Rivaroxaban; BID: bis in die; OD: once daily.

**Table 3:** Results of the study.

Results of the Study			
	Full-Dose DOACs (n = 113)	Reduced-Dose DOACs (n = 72)	*p*
**VTE occurrence/recurrence, n (%)**	0 (0%)	0 (0%)	-
**MB, n (%)**	0 (0%)	0 (0)	-
**CRNMB, n (%)**	3 (2.7%)	3 (4.2%)	0.57
**Villalta Score, mean** ± **SD**	5.24 ± 4.51	5.11 ± 3.73	0.72
**PTS, n (%)**	59 (52.2%)	32 (44.4%)	0.30
**Elastic stockings adherence, n (%)**	101 (89.4%)	66 (91.7%)	0.61

Legend: DOACs: Direct Oral Anticoagulants; n: number; VTE: Venous thromboembolism; SD: Standard Deviation; PTS: Post-thrombotic syndrome; MB: Major bleeding; CRNMB: Clinically Relevant Non-Major Bleeding.

## Data Availability

Datasets generated and/or analyzed during the current study are available from the corresponding author upon reasonable request.
